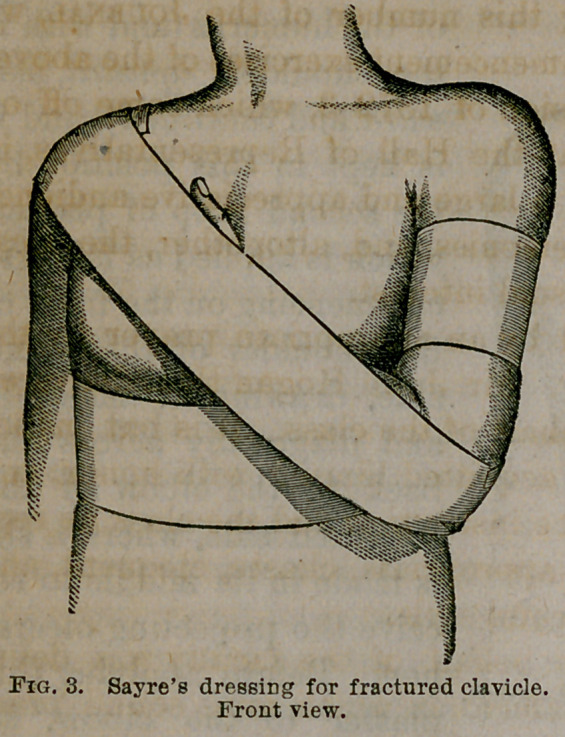# Editorial Correspondence

**Published:** 1873-02

**Authors:** W. F. Westmoreland

**Affiliations:** New York


### EDITORIAL AND MISCELLANEOUS.

### EDITORIAL CORRESPONDENCE.

New York, December 28, 1872.

Dear Journal,—Since my letter of a week or more ago, I have frequently met my old friend, Dr. Lewis A. Sayre, of Bellevue Hospital Medical College, and have seen him perform several operations, among others a resection of the hip-joint for that most terrible affection, hip-joint disease. I was sorry to learn that the Doctor had suffered greatly during the winter with rheumatism of the wrist-joint, which partially disabled him for a time. Although he has not entirely recovered from the attack yet sufficiently so to perform his favorite operation—resection of the hip-joint—with more ease, dexterity and skill than any surgeon than I have ever seen attempt the operation.

The results of Dr. Sayre's resection of the hip-joint are truly wonderful. The statement to the effect that all the essential functions of the hip-joint could be preserved after the complete resection of the head and neck of the femur would, only a few years ago, have been regarded as not only untrue, but absurd; still, Dr. Sayre can present more than one case where the head and neck of the femur were entirely removed, and yet the functions of the hip-joint so perfectly preserved that no one, from seeing them walk, would for a moment imagine that anything serious had ever happened to the limb.

I have several times had the pleasure of hearing the Doctor lecture. I regard him as one of the best teachers in the city. He makes no attempt at oratory, but in a plain and emphatic manner impresses the student with the principles of his department and the details of their application, and frequently in language so apt and striking as to never be forgotten by his hearers. I had the pleasure, yesterday, of hearing him deliver a clinical lecture, at Bellevue Hospital, on fractures of the clavicle, and at the same time saw him illustrate his new dressing for this injury, by its application upon a patient then in hospital. I was so much impressed with his lecture, and his new method of treating fractures of this bone, that I have determined that I could in no other way so much interest your readers as by giving in this letter Dr. Sayre's new dressing for fractures of the clavicle. This I propose to do in his own language, by extracts from a paper published in the *American Practitioner* some months ago. After alluding to the man difficulties in the treatment of this injury—the variety of apparatus, bandages, dressings, etc., that have been suggested to keep the fragments in position—and impressing, by reference to prominent authors, the almost universally admitted fact that there is no successful treatment, or treatment without deformity, he commences the discussion of the displacement in the injury, and the principle he adopts in the treatment, as follows:

"All authors agree as to the deformity which occurs in fracture of the clavicle—viz: that the shoulder falls *downward, forward* and *inward*, and that the outer end of the sternal or inner fragment always rides *over* the inner end of the outer or acromial portion of the clavicle. They also all agree as to the indications to be fulfilled in the treatment—viz: to sustain the shoulder *upward, outward* and *backward*, and to *press* the elevated portion of the clavicle into its proper position.

"My method of keeping the inner portion of the clavicle from riding over the outer portion is *by putting the clavicular portion of the pectoralis major muscle on the stretch*, and compelling it to *pull* the clavicle in place, and thus overcome the tendency of the clavicular portion of the sterno-cleido-mastoid to elevate it, which it will always do unless this precaution is taken.

"M. Guillon (*L'Abeille Medicale*, Oct. 1847,) came nearer the correct method of treatment than any of his predecessors when he recommended placing the arm *behind* the body instead of bringing it over the chest in front; for by this means the *clavicular portion* of the pectoralis major is made very tense, and thus prevents the elevation of the inner portion of the clavicle by the contraction of the sterno-cleido-mastoid. As far as I can ascertain, this is the first attempt to treat fracture of the clavicle by taking advantage of the muscles attached to the bone, and make *them* hold the bone in apposition by keeping them in equal tension on either side of it; but while this position of the arm behind the body drags down the inner fragment of the clavicle to the proper level, it fails to extend the clavicle, and thus permits the pieces to overlap, and also fails to keep the shoulder upward, outward and backward, which is absolutely necessary in order to preserve the fractured portions of the clavicle in accurate apposition.

"I therefore, after drawing the arm backward and retaining it there by a strip of adhesive plaster, pass another piece of plaster over the *well shoulder* across the back, and by pressing the elbow well *forward* and inward, the first plaster around the middle of the arm is made to act as a *fulcrum*, and the shoulder is necessarily carried *upward*, *outward*, and *backward;* and the plaster, being carried over the elbow and forearm (which is flexed across the chest) to the opposite shoulder, the place of starting, and then secured by pins or stitches, permanently retains the parts in position.

"I formerly commenced the first plaster on the inner side of the biceps; but I found that that muscle would roll around and the plaster would lose its hold, requiring to be renewed occasionally; and if it completely encircled the arm for the purpose of stronger attachment, it would arrest the circulation and thus prove dangerous. I have therefore adopted the following plan: Strong and good adhesive plaster (Maw's moleskin is the best) is cut into strips, three to four inches wide (narrower for children); one piece long enough to surround the arm and go completely around the body, the other to reach from the sound shoulder around the elbow of the fractured side and back to the place of starting. The first piece is passed around the arm just below the axillary margin, and pinned or stitched in the form of a loop sufficiently large to prevent strangulation, leaving a portion of the back of the arm uncased by the plaster. The arm is then drawn downward and *backward* until the clavicular portion of the pectoralis major muscle is put sufficiently on the stretch to overcome the sterno-cleido-mastoid, and thus pull the inner portion of the clavicle down to its level. The plaster is then carried smoothly and completely around the body and pinned to itself on the back to prevent slipping, as seen in figure 1. This strip of plaster fulfils a double purpose; first, by putting the clavicular portion of the pectoralis major muscle on the stretch, it prevents the clavicle from riding upward; and secondly, acting as a *fulcrum* at the center of the arm, when the elbow is pressed downward, forward, and inward, it necessarily forces the other extremity of the humerus (and with it the shoulder) *upward, outward* and *backward;* and it is kept in this position by the second strip of plaster, which is applied as follows: commencing on the front of the shoulder of the sound side, drawing it smoothly and diagonally across the back to the elbow of the fractured side, where a slit is made in its middle to receive the projecting olecranon. Before applying this plaster to the elbow, an assistant should press the *elbow well forward and inward* (figure 2), and retain it there, while the plaster is continued over the elbow and fore-arm (pressing the latter close to the chest, and securing the hand near the opposite nipple); crossing the shoulder at the place of beginning, it is there secured by two or three pins, as seen in figures 2 and 3.

FIG. 1. Sayre's first bandage for fractured clavicle. Back view.

"When this has been done the deformity will have entirely disappeared, the fractured bones will be accurately adjusted, and as long as the strips of plaster maintain their position no amount of force can displace them. I have repeatedly tested this fact before my class by seizing the patient by the arm of the fractured side, and whirling him like a top on his feet, without ever caus-

FIG. 2. Sayre's second bandage for fractured clavicle. Back view.

ing the slightest displacement or giving the slightest pain. By this plan of treatment, the patient is only detained from his daily avocation a sufficient length of time to properly adjust the strips of adhesive plaster.

FIG. 3. Sayre's dressing for fractured clavicle. Front view.

"In one instance, a prominent lawyer of this city slipped upon the ice and fractured his clavicle on his way down town. He was brought to my office. I dressed him in the manner above described at nine A.M. and before eleven he was pleading his case in the open court.

"A blacksmith was brought to my office with a fracture of the left clavicle. I dressed it, and in less than an hour he was again working at his forge with his other arm, and continued his labor without any interruption. In both cases the union was perfect and without *any* deformity.

"I could multiply these cases by many similar ones, and I therefore feel quite confident that if any surgeon will follow the plan suggested he will have equally good results."

In the case above alluded to at Bellevue Hospital, I examined carefully the position of the fragments after the dressing, and found them accurately adjusted, with no deformity whatever. The dressing is simple and easily applied, and we bespeak for it a trial at least. Truly,

W. F. WESTMORELAND.

---

DO NOT forget to address "Herald Publishing Company, Proprietors of the Atlanta Medical and Surgical Journal."

MEDICAL WORKS and other publications received will be noticed at an early period.

## Figures and Tables

**Fig. 1. f1:**
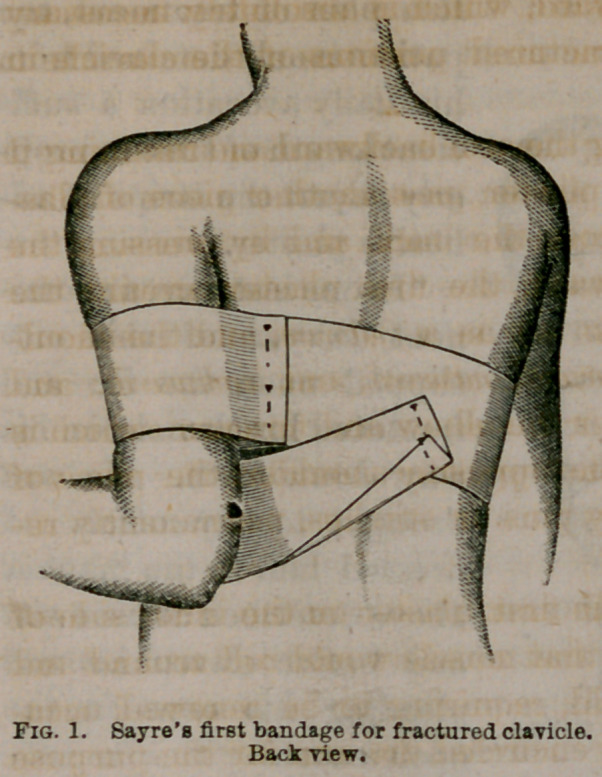


**Fig. 2. f2:**
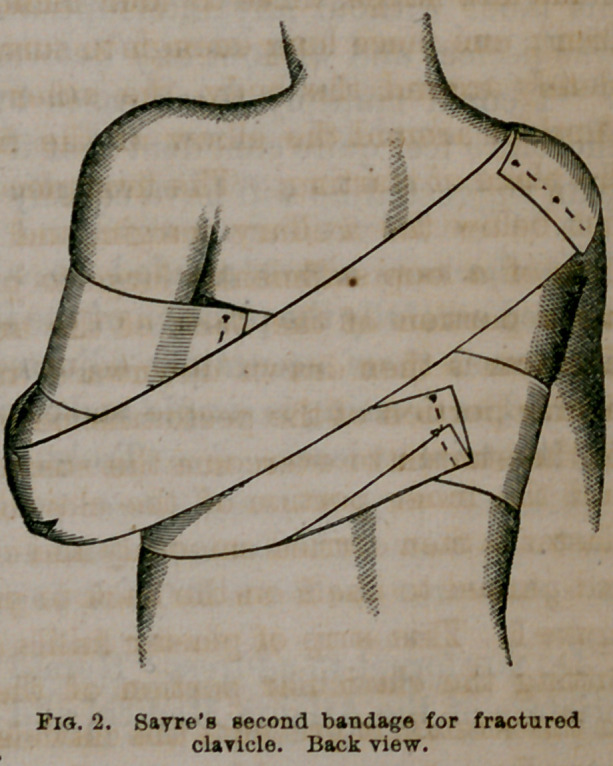


**Fig. 3. f3:**